# Within- and Cross-Modal Distance Information Disambiguate Visual Size-Change Perception

**DOI:** 10.1371/journal.pcbi.1000697

**Published:** 2010-03-05

**Authors:** Peter W. Battaglia, Massimiliano Di Luca, Marc O. Ernst, Paul R. Schrater, Tonja Machulla, Daniel Kersten

**Affiliations:** 1 Brain and Cognitive Sciences and Computer Science and Artificial Intelligence Laboratory, Massachusetts Institute of Technology, Cambridge, Massachusetts, United States of America; 2 Max Planck Institute for Biological Cybernetics, Tübingen, Germany; 3 Department of Psychology, University of Minnesota, Minneapolis, Minnesota, United States of America; 4 Department of Computer Science, University of Minnesota, Minneapolis, Minnesota, United States of America; New York University, United States of America

## Abstract

Perception is fundamentally underconstrained because different combinations of object properties can generate the same sensory information. To disambiguate sensory information into estimates of scene properties, our brains incorporate prior knowledge and additional “auxiliary” (i.e., not directly relevant to desired scene property) sensory information to constrain perceptual interpretations. For example, knowing the distance to an object helps in perceiving its size. The literature contains few demonstrations of the use of prior knowledge and auxiliary information in combined visual and haptic disambiguation and almost no examination of haptic disambiguation of vision beyond “bistable” stimuli. Previous studies have reported humans integrate multiple unambiguous sensations to perceive single, continuous object properties, like size or position. Here we test whether humans use visual and haptic information, individually and jointly, to disambiguate size from distance. We presented participants with a ball moving in depth with a changing diameter. Because no unambiguous distance information is available under monocular viewing, participants rely on prior assumptions about the ball's distance to disambiguate their -size percept. Presenting auxiliary binocular and/or haptic distance information augments participants' prior distance assumptions and improves their size judgment accuracy—though binocular cues were trusted more than haptic. Our results suggest both visual and haptic distance information disambiguate size perception, and we interpret these results in the context of probabilistic perceptual reasoning.

## Author Summary

To perceive your surroundings your brain must distinguish between different possible scenes, each of which is more or less likely. In order to disambiguate interpretations that are equally likely given sensory input, the brain aggregates multiple sensations to form an interpretation of the world consistent with each. For instance, when you judge the size of an object you are viewing, its distance influences its image size that projects to your eyes. To estimate its true size, your brain must use extra information to disambiguate whether it is a small, near object, or large, far object. If you touch the object your brain could use the felt distance to scale the apparent size of the object. Cognitive scientists do not fully understand the computations that make perceptual disambiguation possible. Here we investigate how people disambiguate an object's size from its distance by measuring participants' size judgments when we provide different types of distance sensations. We find that distance sensations provided by viewing objects with both eyes open, and by touching the object, are both effective for disambiguating its size. We provide a general probabilistic framework to explain these results, which provides a unifying account of sensory fusion in the presence of ambiguity.

## Introduction

For well over a century [1],[2] psychologists have considered the question of how the brain uses visual angle sensations to make judgments of an object's size, overcoming the confounding effect of its distance - but the topic remains unsettled. Holway and Boring [3] found that when strong sensations of an object's distance were made available, human size matching performance at different distances was high, but when distance sensations were removed human perception of an object's size was erroneously dominated its visual angle. Epstein et al. [4] surveyed literature regarding the “size-distance invariance hypothesis” [5], which holds that retinal visual angle constrains perception of an object's size and distance such that their ratio holds a constant value (e.g. doubling an object's physical distance while hold its retinal image size constant causes its perceived size to double), and concluded the size-distance invariance hypothesis was subject to a variety of failures. Several studies attributed participants' mistaken size perceptions [4], [6]–[12] to misjudgments of physical distance, while others point out that specific experimental design choices and task demands contribute to reported failures of size constancy [13]–[16]. Recently Combe and Wexler [17] reported that size constancy is stronger when the relative distance between observer and object varies due to observer motion, than when due to object motion. Such findings highlight the unsettled state of current empirical knowledge about human size and distance perception, which is exacerbated by the absence of a unified theoretical account for normative size/distance perception.

We hypothesize that the brain makes size inferences by incorporating multiple sensations based on knowledge of their generative relationship with physical environment properties, and that failures like inaccuracy and systematic biases are due to poverty, unreliability, and/or mistrust, of observed sensations. Our experiments tackle the issue of how the brain incorporates distance information, in particular binocular and haptic (touch), to jointly perceive of how an object's size is changing. Size-change perception, which surprisingly has not been studied in the size/distance perception literature, bears close similarity to static size perception because size-change judgments based on retinal image size are ambiguous if information about the object's motion-in-depth is unknown. However when auxiliary sensations indicating motion-in-depth are available, an observer may rule out size-change/motion combinations that are inconsistent with the auxiliary sensations, and unambiguously infer whether the object is inflating or deflating. We predicted that despite the inherent novelty of the stimuli (i.e. objects do not typically change in size while moving in depth), participants' abilities to discriminate whether an object inflated or deflated would depend on the availability and quality of information about its motion-in-depth. Because binocular and haptic sensations provide information about depth, we predicted that they would each be incorporated for improving size-change judgments. Thus our study answers two key questions: 1) Does the brain use distance-change information for size-change perception? 2) What are the roles of binocular and haptic distance-change information?

**Figure 1:**
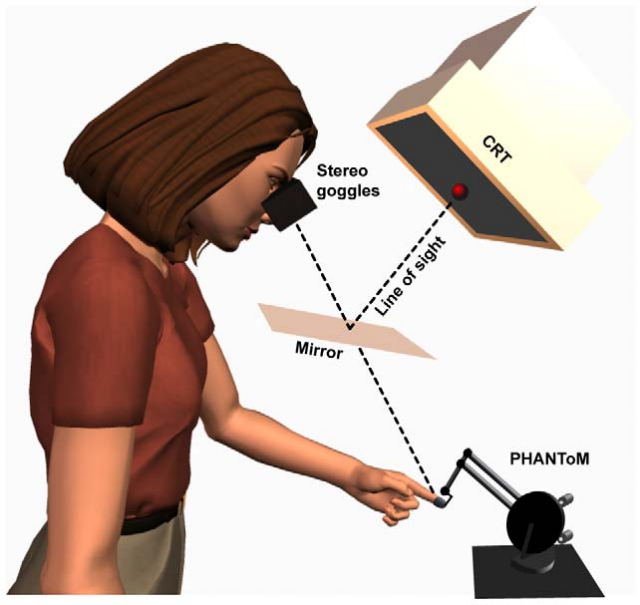
Experimental apparatus. Participants viewed a mirror that reflected the stimulus image from a monitor suspended overhead, such that the image depicted objects located in front of the participants. Participants viewed the mirror through Stereographics stereo glasses that allowed the computer to present stimuli independently to one, or both, eyes. Binocular depth stimuli were achieved by presented different images to each eye that simulated the appropriate stereo disparity. Beneath the mirror, participants' fingertips were attached to a PHANToM (Sensable Technologies) robot arm that allowed the computer to apply forces to the finger simulating rigid surfaces and objects.

**Figure 2:**
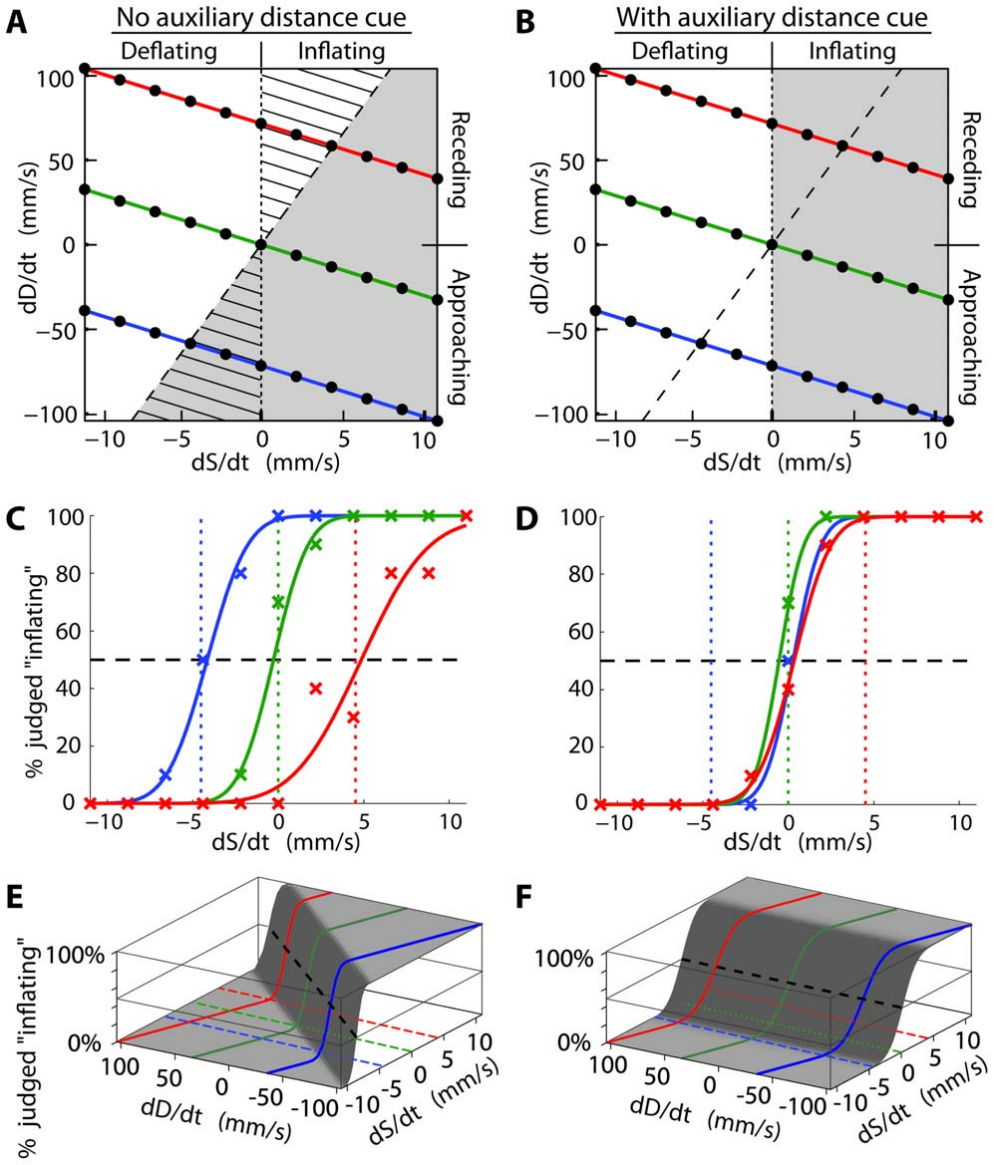
Experiment 1 predictions and data format. A–B: Trial parameters and predictions. The figure depicts the combinations of size- and distance-rates used in different trials, and hypothetical predictions. The x- and y-axes represent the rates of change of a ball's physical size and distance, respectively. Each quadrant corresponds to one combination of approaching/receding and inflating/deflating. Each black dot indicates a pair of distance- and size-change rates presented as a trial during the experiment (each trial was repeated 10 times). The trials' rates were chosen so they fell into 3 distance-direction groups (colored line lines), approaching (blue), receding (red), and intermediate (green). The diagonal, dashed line in A and B is a discrimination boundary representing size- and distance-rate combinations that would result in zero image size-change. The vertical dotted line in A and B is a discrimination boundary representing zero physical size-change. An observer who relies fully on the ball's changing image size, (e.g. the “ambiguous” H−/B− cue condition), would judge the ball to be “inflating” for trials to the right of the discrimination boundary (shaded region of Panel A), and make errors for stimuli that fall in the triangular hatched regions. An observer who correctly uses the distance cue(s) (e.g. the “unambiguous” H+/B+ condition) would completely disambiguate size and distance and make “inflating” size judgments (shaded region of Panel B). C–D: Psychometric functions for H−/B− (C) and H+/B+ (D) distance-cue conditions (participant 5). Each graph depicts the proportion of trials judged “inflating” in the approaching (blue), intermediate (green), and receding (red) distance-direction groups, for participant 5. The x-axis represents size-change rate (mm/s) and the y-axis represents the percent of trials judged “inflating”. The ‘X's represent actual data and the curves represent best-fit psychometric functions (cumulative Gaussian). The horizontal gray lines represent points at which the ball would be judged as “inflating” 50% of the time. The vertical colored dashed lines indicate the size-change rates that correspond to zero image size-change for each distance-change direction condition (the intersections of the diagonal dashed line with the colored lines in Box A). E–F: 3D psychometric functions for H−/B− (C) and H+/B+ (D) distance-cue conditions (participant 5). The surface plots depict participant 5's choice probabilities for the H−/B− and H+/B+ conditions. The x-axis represents size-change rate, the y-axis represents distance-change rate, and the z-axis represents the percentage of trials in which the participant judged the ball as “inflating”. The curves are schematic, they represent the average psychometric function estimates across the three distance-cue conditions, interpolated so that the PSEs lay on the discrimination boundary. The colored lines overlaid on the surfaces are similar to those in boxes C–D. This figure shows the relationship between the psychometric functions in boxes C–D and the participant's associated “inflating” size judgments shown in boxes A–B. The heavy black dotted line corresponds to the confusion (white-gray boundary in boxes A–B).

Our size-change discrimination task (Figure 1) presented participants with an object that either inflated or deflated while simultaneously either approaching or receding, and asked them to discriminate whether it inflated or deflated (Figure 2). Most static size perception tasks use matching paradigms, and our task was advantageous because it allowed us to present a single stimulus per trial, and avoid issues regarding relative comparison of pairs of stimuli. We provided participants with different types of auxiliary motion-in-depth information, binocular [3],[16],[17] and haptic [18],[19], both in isolation and simultaneously, and examined their inflation/deflation judgments to evaluate how auxiliary distance information influenced perceived size-change. Evidence for the use of binocular and haptic distance information in size-change perception has not been reported, and previous studies of cue integration [20] suggest the brain combines haptic and binocular information in proportion to its reliability to jointly improve spatial perception.

We found that when distance-change information was absent, participants' size-change judgments closely matched object's image size-change. However, when we provided participants with auxiliary distance-change sensations, participants incorporated this additional information to form more accurate size percepts that were consistent with both monocular *and* auxiliary sensations. Moreover when both binocular and haptic information was presented, most participants showed greater disambiguation of size than when either was presented in isolation. These results suggest size-change perception uses knowledge of how multi-modal size and distance sensations are related to interpret the scene. We interpret these findings in the framework of probabilistic perceptual inference, in which available sensations are combined according to their relationship to scene properties and their respective reliabilities [21],[22].

## Results

### Experiment 1: Distance disambiguation for size perception

Experiment 1 contained four *distance-cue conditions* (H−/B−, H+/B−, H−/B+, H+/B+) that provided the four possible combinations of the presence (+) or absence (−) of haptic (H) and binocular (B) cues to the ball's distance-change. Haptic cues include proprioceptive and pressure information generated by the ball's movement in depth, and binocular cues include vergence and relative retinal disparity information that gives direct information about the ball's trajectory (see Methods and Text S1). Figures 2A–B show grids on which we plot the ball's size- and distance-change rates for all stimuli (black dots). The diagonal dashed line divides the stimuli into those in which the ball's *image size* increases (lower-right) versus decreases (upper-left) in size, and the vertical dotted line divides the stimuli into those in which ball's *physical size* inflates (right) versus deflates (left).

Our specific analysis and results are as follows. We separated balls' distance-change rates into three *distance-direction* groups: *receding*, *intermediate*, and *approaching* (colored lines, Figures 2A–B). For each group we fit individual psychometric functions (cumulative Gaussian), where the height of the function at a particular size-change rate indicates the percentage of trials the participant judged “inflating”. Figures 2C–D depict the results for one participant corresponding to the distance-direction group in Figures 2A–B. Figures 2E–F illustrates the relationship between the psychometric function fits and the shaded regions in Figures 2A–B. Within each distance-cue condition, we found each psychometric function's 50% point, and fit a line between these points. We termed these best-fit lines participants' *discrimination boundaries* between “inflating” and “deflating” responses, and interpreted them as measures of participants' *confusion*. Specifically, we computed the best-fit slope with respect to distance-change rate (y-axis), and normalized it into a *confusion ratio*. A confusion ratio of 1 meant the participant discriminated inflation from deflation depending exclusively on the sign of the image size-change rate, which corresponded to the locus of physical distance- and size-change rates that produced an image-change rate of 0 (diagonal line, Figure 2A). A confusion ratio of 0 meant the participant discriminated inflation from deflation depending on the sign of the physical size-change rate (vertical line, Figure 2B). Simply put, when a participant's discrimination judgments were independent of the nuisance distance property they did not confuse distance-change for size change (zero confusion), and when their discrimination judgments were dependent on the nuisance distance property they confused distance-change with size change (confusion of 1). Our “confusion ratio” is related to the Brunswick and Thouless ratios, which apply to static size matching tasks [23]. Notably, those ratios scale inversely to ours: they take values of 1 when participants comparison size judgments match the standard stimulus size (confusion of 0), and 0 when the comparison size judgment matches the image size (confusion of 1).

**Figure 3:**
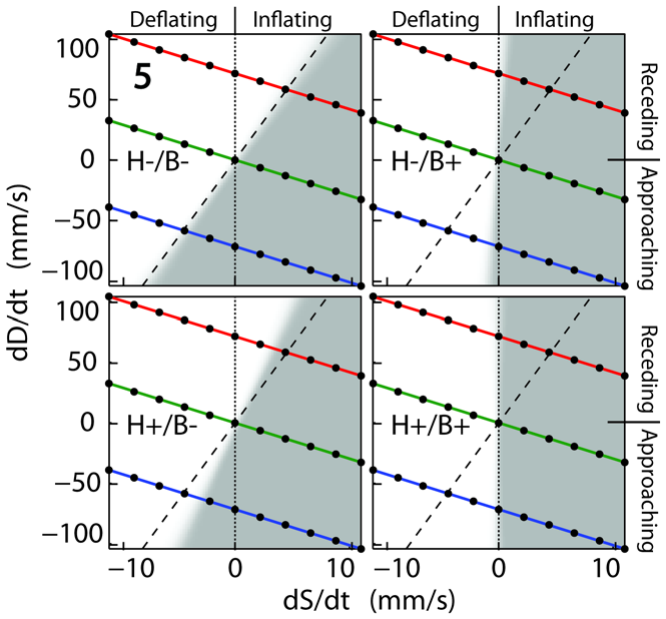
Experiment 1 discrimination boundaries (participant 5). This figure depicts participant 5's discrimination boundaries in all distance-cue conditions, on the same axes as in Figures 2A–B. Each box is a single distance-cue condition (indicated by “H*/B*” on left side of each box). The colored lines are the same as those depicted in Figure 2. Gray regions represent size- and distance-change combinations predicted to be judged “inflating” more than 50% of the time by discrimination boundary fit to participants' PSEs; white regions represent combinations predicted to be judged “deflating” more than 50% of the time.

**Figure 4:**
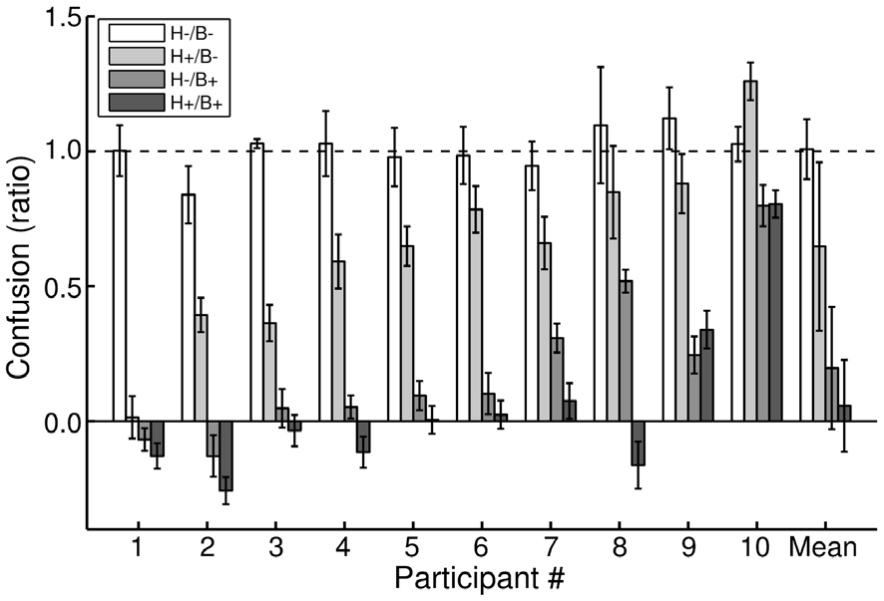
Experiment 1 size-change confusion. The figure depicts the size-change confusion for each participant, and the group mean. Each bar is a single distance-cue condition's size-change confusion, with 1 MADC error bars (can be interpreted similarly to standard error, see Data Analysis). The distance-cue condition is indicated by the bar's shading and referenced in the legend. The horizontal dashed line indicates the predicted confusion for an observer that relies exclusively on the image size-change cue to make physical size-change judgments; this is why the H−/B− condition bars, in which only image size-change cues were available, all overlap the horizontal dashed line.

In the trials that contained no distance cues (H−/B−), we predicted participants would rely on prior assumptions that the ball tends to stay still (or move slowly). This is a sort of motion analog to the “specific distance tendency” [10]. Slow movement priors have previously been reported for 2D motion perception [24]–[26] and others [27] find similar priors in 3D [28]. Assuming slow, or no, movement would bias participants to attribute increasing image size largely to inflation and in turn lead them to judge stimuli with increasing image sizes as “inflating” (shaded grey in Figures 2A–B). All participants display precisely this pattern; Figure 3 (top-left box) shows the specific pattern for a typical participant (5) in the H−/B− condition, and Figure 4 summarizes all participants (white bars). The evidence suggests that participants used prior assumptions that objects tend to stay at rest to disambiguate the scene. But because the ball was often approaching or receding, these often-incorrect prior assumptions led to erroneous perceptual size judgments. However, if we had allowed participants to decide whether the ball was changing size or changing distance, they may have preferred changing distance in some cases - it may be that the role of the prior is guided by the task's demands.

In those conditions that contained auxiliary distance-change cues (H+/B−, H−/B+, H+/B+), we predicted participants would perceive trials with increasing image size as “inflating” (shaded regions in Figure 2B) only when the ball's movement in depth could not account for the changing image size; in other words, the participant will not perceive a rapidly approaching ball as inflating if the image size is only increasing a small amount. Likewise, when the ball's image size was decreasing, we predicted participants would perceive the ball as deflating only when the recession rate was not great enough to account for the image size change. All participants exhibited this pattern when the auxiliary binocular cue was present (H−/B+ and H+/B+), and 7 of 10 also showed size disambiguation when the haptic cue alone was present (H+/B−); again, Figure 3 (bottom-left, and right boxes) shows the specific pattern for a typical participant (5) in the H+/B−, H−/B+, and H+/B+ conditions, and Figure 4 summarizes all participants (grey bars). These results indicate that participants disambiguate the scene using both haptic and binocular distance-change cues, by augmenting their prior assumptions to make more accurate inflation discriminations.

Figure 4 presents confusion for all participants in all distance-change conditions. A two-way, repeated-measures ANOVA found a significant reduction of confusion across participants for both haptic (F(1, 9) = 17.42, p<0.005) and binocular (F(1, 9) = 212.5, p<0.0001) distance-change cues, and no significant interaction (F≈0, p>0.05) (though the fact that the binocular cue almost fully disambiguated the inflation/deflation rate for most participants means any interaction effect would be masked by the ceiling).

Our results indicate participants use binocular distance-change cues significantly more than haptic cues for disambiguating the scene and improving physical size judgments (H+/B− vs. H−/B+ conditions compared in a paired sign test, p<0.002). Previous cue combination studies [20], [29]–[38] have demonstrated integration of cues in proportion to their relative reliabilities. If each auxiliary cue, binocular and haptic, was trusted by the observer to provide information about the ball's distance-change, we hypothesized that their disparate explaining-away effects were due to the binocular cues' greater reliability over the haptic cues'. We examined whether Experiment 1's binocular/haptic discrepancy was due to differences in haptic and binocular cue reliabilities in Experiment 2.

### Experiment 2: Distance-change cue reliability

We measured the haptic and binocular cues' noise (see [39]) to determine whether differences in their respective reliabilities could explain their discrepant effects on disambiguating the balls' inflation/deflation rates in Experiment 1. Participants observed two moving balls sequentially, and judged which ball moved faster, in a two-interval forced choice (2IFC) discrimination task. Experiment 2 used binocular and haptic cues in different conditions, so we could measure their respective reliabilities in isolation. The ball's movements were always restricted to the depth axis (with slight fronto-parallel oscillation described in the Methods) as in Experiment 1, and also spanned the same speed range as Experiment 1. In the haptic condition, the ball was not visible during the stimulus interval; in the binocular condition the ball was visible and its image size changed under accurate perspective projection (see Methods for details).

**Figure 5:**
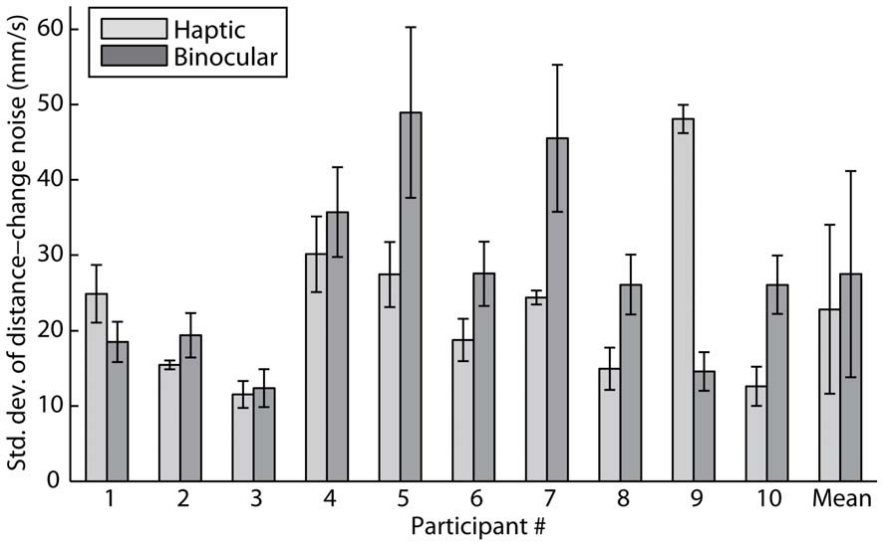
Experiment 2 distance cue noise standard deviations. The figure depicts the inverse-reliability of the haptic and binocular distance-change cues for each participant, and pooled across all participants. Each bar represents the standard deviation of the noise that corrupts a distance-change cue, with 1 MADC error bars (see Data Analysis). The haptic cue is indicated by the light bars, the binocular cue by the dark bars.

Our results show that with the exception of one participant, the haptic and binocular cue reliabilities do not explain their differential uses in Experiment 1. Figure 5 shows the haptic and binocular distance-change noise magnitudes for each participant, where each pair of bars represents the haptic and binocular noise magnitudes (standard deviation) for a participant. Qualitatively it is clear that the binocular and haptic noises have comparable magnitudes. By comparing the set of bootstrap-resampled binocular and haptic noise magnitudes, we can perform a hypothesis test of the prediction that the binocular noise is less than the haptic noise. All participants fail this test (p>0.05), except participant 9 (p<0.05). Thus, differences in cue reliabilities cannot explain Experiment 1's discrepant use of binocular and haptic cues to reduce confusion. This effect is consistent with the observer trusting the binocular cue more greatly than the haptic, thus integrating less of the haptic cue information.

## Discussion

Our study finds that humans use within- (binocular) and cross-modal (haptic) distance-change sensations to disambiguate otherwise ambiguous monocular image size sensations, resulting in more accurate judgments of object size. Binocular distance-change cues influenced participants' size judgments more strongly than haptic cues. When both modalities' distance-change cues were presented simultaneously, nine of ten participants' physical size judgments were virtually confusion-free.

**Figure 6:**
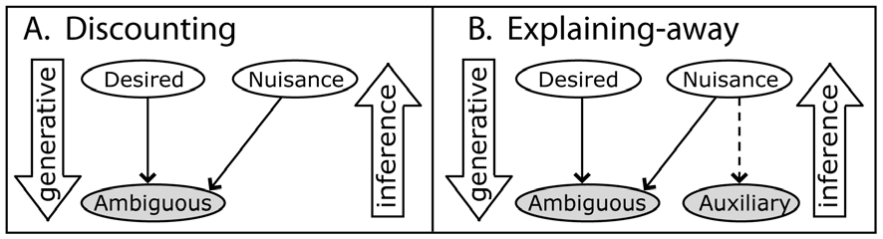
Bayesian inference: from discounting to explaining-away. Perception is characterized by two complementary processes: 1.) The “generative process” determines how scene properties, such as an object's physical size and distance, cause the observer's sensations, such as monocular image cues, binocular, and haptic information, and 2.) The scene “inference process” characterizes the observer's use of generative and prior knowledge to recover local scene properties. The generative process can be summarized by a conditional likelihood 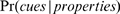, the inference process by the posterior probability distribution, 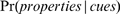. Bayes' rule dictates how each process relates: 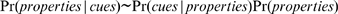, where 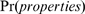 represents the prior probability distribution over scene properties. In the figures above, scene properties are represented by white nodes, and cues are represented by gray nodes. In our experiment, the desired property was the physical ball size, the nuisance property was the physical ball distance, the ambiguous cue was the monocular image size cue, and the auxiliary cue was provided by the binocular and haptic distance cues. A.) Discounting inference: a desired property influences a single cue, which is ambiguous due to the confounding influence of a nuisance property. The single ambiguous cue can be used to estimate the desired scene property that caused it by discounting the effect of the nuisance property using prior knowledge about it. The conditional relationships (arrows) in Box A specify that Bayes' rule can be factored such that:
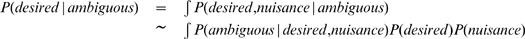
B.) Explaining-away inference: similar structure to discounting, but involves additional, auxiliary cues. By using the auxiliary cue to “explain-away” the influence the nuisance property has on the ambiguous cue, the desired property can be unambiguously inferred. Bayes' rule specifies inferring the desired property as:
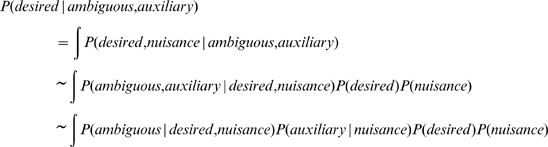
The ambiguous and auxiliary cues can be factored because they are conditionally independent given the nuisance property.

In order to use the distance-change to improve size-change judgments, the brain must use generative knowledge of how an object's physical size and distance cause monocular image size- and distance-change cues to alleviate the confounding effects of physical distance-change. Such knowledge may be abstractly represented (the laws of physics) or encoded in a more applied manner (a look-up table relating size, distance, and image cues). This is consistent with a core feature of Bayesian reasoning termed *explaining-away* [40]. Knowledge about the relationships between world properties and sensations provides perceptual inference processes with a common representation for integrating prior knowledge with sensory evidence, and probabilistically “solving for” scene properties based on sensations. Bayesian reasoning as a framework for interpreting perceptual behavior has attracted considerable attention because it provides a principled theoretical framework for describing the brain's recovery of scene properties from sensations [22],[41] and has allowed quantitative confirmation that humans exhibit near-optimal perceptual performance across many tasks [20], [22], [29], [33], [35]–[38]. Various studies have found that when humans judge single scene properties that produce multiple pieces of sensory information, or *cues* (Figure 6A), they average the cues in proportion to their reliability [25], which is the Bayes'-prescribed perceptual strategy. Others report [42]–[44] perceptual “discounting”, in which prior knowledge is used to disambiguate otherwise ambiguous sensory cues, which requires knowledge of the generative relationship between a cue and the scene properties that cause it.

Our study examines a more complex situation (Figure 6B) where, unlike discounting [42]–[44] (Figure 6A), correct inference of the *desired* scene property (physical size-change) requires an inference strategy that exploits generative knowledge of the relationships between *multiple* scene properties (physical size-change and physical distance-change) and *multiple* sensations (retinal image size-change, binocular and haptic distance-change cues). No single sensation alone, retinal image size-change or distance-change cue, constrains the physical size-change inference uniquely due to the confounding influence of *nuisance* scene properties – properties that affect sensations but do not contribute to the judgment - in this case, physical distance-change (Figure 6B). Because the nuisance property (physical distance) confounds the direct cue (retinal image size-change) to the desired property (physical size-change), incorporating *auxiliary* cues (distance-change sensations) can explain-away the influence of the nuisance physical distance-change and allow unambiguous judgments of physical size-change.

Explaining-away can characterize other perceptual tasks in which multiple scene properties influence multiple cues in the manner depicted by Figure 6B; for example, estimating surface reflectance from sensed lightness despite the confounding influence of illumination [45], estimating object shape from image contours despite the confounding influence of pose, and the general class of “perceptual constancy” effects. Also explaining-away is a general Bayesian perspective on a specialized concept [46] termed “cue promotion” - in which a relative cue (like stereoscopic disparity) is able to be incorporated into perceptual judgments (promoted) only because a second, auxiliary cue (like depth from vergence) provides information to make it an absolute cue. Many unimodal perceptual phenomena are characteristic of explaining-away [3], [47]–[48]. Multimodal perceptual explaining-away is less documented, but explaining-away in bistable percepts has been reported [49]–[52] as well as in continuous percepts [53]–[54]. Our results extend previous reports of explaining-away to include continuous, multimodal scene property judgments [47], [50]–[51], [53], [55]–[56].

Explaining-away is only appropriate when the auxiliary cues are dependent on scene properties that influence cues to the desired scene property. This typically occurs when the nuisance variable causes the auxiliary cue. There is evidence suggesting that non-visual sensory cues are integrated less efficiently than their reliabilities afford [33] or in a less committed, reversible manner [39], [57]–[58], and some have attributed lack of cue integration to weak conditional dependency between cues and world properties [31], [58]–[62]. *Reliability* reflects the quality of a cue; if the sensory signal is corrupted by noise the reliability decreases. *Trust* reflects the degree to which the observer believes the cue is related to the desired scene property; there may be other scene properties that influence the auxiliary cue which diminishes the cue's diagnosticity for the desired scene property. In cases in which all auxiliary cues are trusted equally, they should be integrated in proportion to their relative reliabilities only. However, if trust in the auxiliary cues is unequally distributed they should integrated in proportion to the relative reliabilities *and* their trust.

Previous studies that tested multisensory disambiguation of bistable stimuli reported mixed results [50]–[51]. It is possible that these different findings are due to non-visual cues being trusted less due to their frequent independence from visual cues. Alternatively the mixed results may be due to variable cue reliabilities [63], for instance when visual cues to a bistable stimulus's structure vary in relative reliability compared with tactile cues, tactile cues may influence perceived structure in proportion to their reliability. Our experiment was sensitive to partial disambiguation, because participants discriminated percepts that lied on a continuous axis (rate of distance-change), which may reconcile previous mixed results by demonstrating the graded roles of auxiliary cue information. We found different effects of individual haptic and visual cues, and strongest influence when both were present, which argues for the reliability-weighted integration of that information.

One potential reason that binocular distance-change cues were more useful than haptic cues for disambiguating size perception in our experiment may be that the haptic cue is more weakly coupled with the image cue than the binocular cue, perhaps reflecting the causal structure of the world. In decoupled situations, in which different world properties influence different cues independently, it is inappropriate to combine cues. For instance, in natural settings binocular depth and monocular image size cues are transmitted to the eyes by the same light patterns, thus are usually highly dependent. Because, sensory channels for visual and haptic information differ, and there are many situations in which the felt position of an object differs from its visual position, like manipulating a tool, playing with a yo-yo, or touching an object that is occluded by a nearer object. In our experiment the haptic cue was somewhat atypical, because we forced the fingertip to always be positioned at the *center* of the ball, not the edge, so the size-change would not be directly measurable by radial pressure toward or away from the ball's center. It is plausible that this atypicality degraded participants' belief that haptic and visual cues were caused by the same object. Recent reports of visual-auditory cue integration have found causality-modulated cue integration [59]–[62], and it may explain why the haptic cue is trusted less for disambiguation compared with the disparity cue in our experiment.

Another possibility derives from the brain's algorithm used to compute the size-change rate. Per Rushton and Wann ([64], Figure 1 caption), the B+ conditions allow the possibility of estimating the size-change rate without explicitly estimating the distance-change rate (by computing the ratio between image-size-change and binocular vergence angle-change rates, which causes the explicit distance-change rate terms to cancel). This means that a potential source of noise in the B+ conditions, incurred during estimation of the distance-change rate, would be removed, allowing higher fidelity disambiguation of the size-change rate in those conditions. If this were the case, Experiment 2 may have overestimated the effect of noise in Experiment 1's B+ conditions depending on how noise enters the system: if noise only corrupts the brain's estimates of binocular vergence angle-change rates, then Experiment 2's binocular noise estimates are valid. However, if noise additionally corrupts the ability to make binocular distance-change judgments, then Experiment 2's binocular noise estimates would be overestimates of the true noise afflicting Experiment 1's B+ conditions. This logic may be moot if the distance-change is used to drive oculomotor vergence dynamics (i.e. tracking in depth) because in that case the noisy distance-change rate would influence the binocular vergence-change rate. Either way, in order to apply the ratio algorithm [64] for computing size-change still requires the brain to understand the generative relationships among size, distance, and the image and binocular sensory cues, which does not diminish our findings.

One future challenge is directly assessing what prior assumptions the perceptual system has about the world, and how reliability and trust in various cues are learned [63]. With quantitative estimates of prior assumptions, one can predict how reliable auxiliary cues must be and how much they should be trusted, to override conflicting priors. Other studies [5] refer to a “specific distance tendency” in which participants assume objects appear at a canonical distance. In the 2D motion perception domain and [24],[26] each reported that humans exhibit strong prior preferences for “slow and smooth” movement, and our study suggests participants assume objects move slowly in 3D, but a stronger direct test of 3D motion priors requires quantitative predictions. Measuring prior knowledge directly is difficult, but developing indirect methods is an important topic of recent and continuing research [26].

Our results indicate that the brain uses multisensory distance-change cues to improve perceptual size-change disambiguation. Haptic and binocular distance-change cues are both effective, binocular more than haptic, which is not explained by their relative reliabilities, but is consistent with causal cue integration models [61]–[62]. Our findings support the view that perceptual processing employs knowledge of the sensory generative process to infer scene properties and disambiguate competing interpretations.

## Methods

### Ethics statement

Experiments were undertaken with the understanding and written consent of each subject, with the approval of the Ethik-Kommission der Medizinischen Fakultät und am Universitätsklinikum Tübingen, and in compliance with national legislation and the Code of Ethical Principles for Medical Research Involving Human Subjects of the World Medical Association (Declaration of Helsinki).

### Participants

11 right-handed participants (ages 18 to 35) with normal/corrected-to-normal vision (Snellen-equivalent of 20/25 or better) and normal stereopsis (60 s of arc or better - Stereotest circles; Stereo Optical, Chicago) were recruited from MPI Tuebingen's Subject Database and compensated 8 €/h. All participants completed both Experiments 1 and 2, with the exception of one who was excluded from reported results because her responses indicated she did not follow the experimenters' instructions.

### Apparatus

Participants sat in a virtual workbench that presented both graphical and haptic stimuli (Figure 1; see [20] for details). Participants' heads were stabilized with a chin-and-forehead rest 45 deg forward. Visual stimuli were presented on a monitor (21″ GDM-F500R SONY, 38.2×29.8 cm, resolution of 1280×1024 pixels, refresh rate 100 Hz) whose center was 50 cm from the eyes reflected on a first-surface mirror, and whose top was tilted 22 deg backwards from the fronto-parallel plane. Binocular stimuli were presented through CrystalEyes TM (StereoGraphics) liquid-crystal shutter glasses which allowed different images to be presented to each eye. Haptic stimuli were presented using a Premium PHANToM force-feedback device (SensAble Technologies), to which the index finger was attached by a thimble and elastic band, allowing six degrees of freedom movements. The 3D fingertip position was monitored continuously, and the computer applied simulated normal forces when the tip reached the positions of the virtual haptic objects. The apparatus was calibrated to spatially align the visual and haptic stimuli, simulating a single scene.

### General procedure

There were two experiments, *1. Distance cue disambiguation for size perception* and *2. Distance cue reliability*, that each contained *haptic* and *binocular* distance cues. At the start of each trial, a 35 mm diameter red ball was placed between 443 mm and 455 mm from the observer (4.4–4.5 deg visual angle). In trials containing a binocular distance cue, the ball was presented binocularly to the observer's two eyes, rendered to simulate an interocular distance of 58 mm. The participant signaled he or she was ready to begin the trial by reaching and contacting the ball with the index finger (attached to the PHANToM device). Once contact was made, the PHANToM device applied forces to the fingertip to guide it to the center of the ball.

At this point the experimental phase of the trial began: the ball began moving in depth with respect to the participant, while simultaneously changing in size, for a duration of 1000 ms. If the trial contained a haptic distance cue, as the ball moved appropriate forces were applied to the fingertip to maintain its position at the center of the ball; otherwise no forces were applied to the fingertip once the ball began to move and participants typically held their fingertips at a roughly constant position. The ball also slightly oscillated in the observer's fronto-parallel plane following a sinusoidal displacement (with amplitude between 5.0 and 15.0 mm) in a random direction and at a random frequency (between 0.35 and 0.5 Hz). This was intended to both decrease the similarity of the visual and haptic trajectories across trials, increase their perceptual fusion, as well as obviate local edge motion information as a direct indicator of image size-change.

Although fixation was not precisely controlled or monitored, our experience and observations of participants suggested they fixated the ball in monocular and binocular conditions. Also, our stimuli were constructed to eliminate two potential sources of size-change information from binocular cues. One source is “Da Vinci” stereopsis, which refers to depth information that results from points on the object that are visible in only one eye due to object self-occlusion. This cue requires identifying object points without correspondences between the eyes. Because the ball has no horizontal luminance/color contrast, Da Vinci stereopsis was eliminated as a cue to size-change. A second potential source of binocular size-change information was disparities due to the ball's oscillation. For a ball in the mid-sagittal plane there are no binocular disparity cues to size change. We determined that the slight oscillatory movements the balls made out of the mid-sagittal plane created sub-threshold (undetectable) relative disparity cues to ball size. See Text S1l for an in-depth examination and schematic of the binocular cue. Lastly, accommodation was a potential cue, uncontrolled except that the screen depth was fixed.

After 1000 ms, the ball disappeared. In Experiment 1, only a single stimulus interval was presented. In the Experiment 2, two stimulus intervals were presented; following the first interval a new ball appeared and the second interval proceeded just as the first. Once the stimulus interval(s) were finished, two buttons appeared on the left side of the scene and participants were instructed to press the button that corresponded to his or her judgment of the scene. The trial ended once the button was pressed, and the subsequent trial began immediately.

In Experiment 1 the buttons were labeled “inflating” and “deflating”, and the participant pressed the button corresponding to his or her perception of the ball's physical size change. We interpreted participants' choices as their discriminations of the ball's absolute size-change rate.

In Experiment 2, each trial was designed as two-interval forced-choice (2IFC). In every trial, both balls moved in the same direction with respect to the participant (approaching/receding), but their speeds were different relative to each other. Also, the balls never changed in size (equivalent to 0 mm/s size-change rate in the main experiment). In haptic trials, the ball disappeared from view as soon as it began to move. Following the two intervals participants were instructed to press one button among two choices, labeled “1st” and “2nd”, indicating which interval contained the faster ball.

### Design specifics

#### Experiment 1

Four distance-change cue conditions were run, distinguished by the type(s) of distance cues that were presented: no-haptic/no-binocular (H−/B−), haptic/no-binocular (H+/B−), no-haptic/binocular (H−/B+), and haptic/binocular (H+/B+). The haptic and binocular distance cues are described above in the General Procedure subsection; each provided a compelling sensation of the ball's changing distance.

The ball's movement rate was selected from between −104.0 and 104.0 mm/s, where a negative velocity corresponds to the ball moving toward the observer and a positive velocity corresponds to the ball moving away, in the line of sight of the participant. Specifically, we used 3 pedestal distance-change rates, {−71.5, 0.0, 71.5 mm/s}, and varied the distance-change around these pedestal values by adding satellite values {−32.5, −26.0, −19.5, −13.0, −6.5, 0.0, 6.5, 13.0, 19.5, 26.0, 32.5 mm/s}, for a total of 33 possible distance-change values. The ball spanned 7.7 deg visual angle at its nearest/largest state and 2.5 deg at its farthest/smallest.

Concurrent with the ball's distance change, its size changed at a rate selected from between −11.0 to 11.0 mm/s, where negative rates correspond to the ball deflating and positive rates correspond to the ball inflating. For each pedestal distance-change, we paired each of the satellite distance-change values with a particular size-change rate from the set {−11.0, −8.8, −6.6, −4.4, −2.2, 0.0, 2.2, 4.4, 6.6, 8.8, 11.0 mm/s}. The pedestal distance-change rates defined which *distance-direction group* (approaching, receding, intermediate; indicated by the line colors in Figures 2C–F) the trial belonged to. In total there were 33 unique distance and size-change rate pairs, each repeated 10 times. Figures 2A–B plots all unique distance- and size-change rate combinations (black dots) as 2D coordinates.

#### Experiment 2

Two conditions were run, haptic and binocular. The experiment was 2IFC and the two intervals were called the standard and comparison, the order in which they were presented was randomly selected before each trial. For each distance-cue condition, two standard distance-change rates were used, {−55.0, 55.0 mm/s}. The comparison distance-change rates differed from the standard by a value from the set {−54.0, −36.0, −18.0, 0.0, 18.0, 36.0, 54.0 mm/s}. Each possible standard and comparison pair was repeated 14 times.

### Data analysis

All confidence intervals were estimated by nonparametric bootstrapping [65], comparable to those used by [66]–[67]. Error bars on some figures were computed using the “median absolute deviations with finite sample correction factors” (MADC) from the LIBRA Robust Statistics toolbox for Matlab [68]. MADC approximates standard deviation estimates of the mean of the sample for normally-distributed data, but it is more robust for skewed and kurtotic distributions.

#### Experiment 1

Maximum-likelihood estimation (MLE) was used to fit participants' size-change discrimination performance with psychometric functions (robust cumulative normal functions, see [66]–[67]) with size-change rate on the abscissa and frequency of responding “inflating” on the ordinate. The Point of Subjective Equality (PSE) was the 50% point on the fitted psychometric functions (horizontal gray lines in Figures 2C–D). Across distance-change directions, *approaching*, *intermediate*, and *receding*, we maximum-likelihood-fit *discrimination boundary* lines to the PSEs to separate ‘inflating’ from ‘deflating’ responses (Figures 2E–F). The free parameters for estimating discrimination boundaries were slope and intercept (with respect to the distance-change axis). Because all error bars were estimated by bootstrapped resampling, if the linear fits were poor models this was represented as increased error bar magnitudes.

We defined the *confusion* as the slope of the discrimination boundary with respect to the distance-change axis; confusion of 1 corresponds to the image-only discrimination boundary (Figure 2A–B diagonal dashed line), while confusion of 0 corresponds to the veridical size-change discrimination (Figures 2A–B vertical dotted line).

#### Experiment 2

We MLE-fit discrimination performance with robust cumulative normal functions [66]–[67] and interpreted the fitted just-noticeable-difference (JND) as 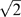 times the standard deviation of the noise which corrupted a single distance-change cue [39]. Each single-cue standard deviation, which we refer to as noise, was an estimate of how reliable each distance-change cue was (reliability is inversely proportional to the noise's variance).

## Supporting Information

Text S1 Details regarding the binocular stimuli presented to participants.
(0.24 MB PDF)
