# Supplementary material for: Within- and Cross-Modal Distance Information Disambiguate Visual Size-Change Perception
Source: PLoS Comput Biol. 2010 Mar 5;6(3):e1000697. doi: 10.1371/journal.pcbi.1000697 (PMC2832682; doi:10.1371/journal.pcbi.1000697)
Supplement: Text S1 — Details regarding the binocular stimuli presented to participants. (0.24 MB PDF) [file pcbi.1000697.s001.pdf]

Supplementary material S1

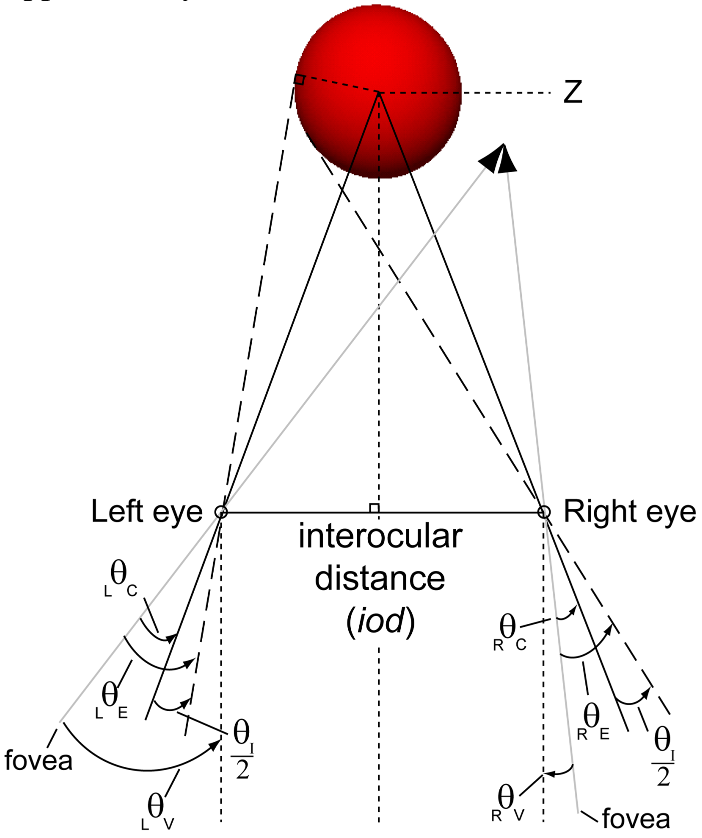

Supplementary material S1: (see text for details)

As stated in the Methods section, the ball was a red sphere on a black background, with no internal contrast features (except a slight top-to-bottom shading gradient to enhance the percept of a 3D object). Thus, the only locations on the stimuli suitable for solving the stereo correspondence problem between the two eyes are at the depth edges between ball and background. In Figure A, notice that the left ball edges are different in each eye's image, but remember that with no contrast features the observer has no information to suggest the correspondences are not co-spatial. Moreover, the maximum possible visual angle of the ball was 7.7 deg, meaning that the maximum amount of this “Da Vinci” stereopsis effect was merely 2% of the ball’s total circumference.

In Figure A, we assume the observer fixates some point in space, indicated by the arrows, by orienting the eyes at angles $\{ {}_L\theta_V, {}_R\theta_V \}$, with respect to the eye’s midline (positive angles mean the vergence is toward the right of that eye’s midline). The observer makes two binocular measurements for the ball at any time, the visual angles of the ball’s center with respect to a fovea for each eye $\{ {}_L\theta_C, {}_R\theta_C \}$, and the left edge of the ball with respect to the fovea for each eye, $\{ {}_L\theta_E, {}_R\theta_E \}$ (this analysis applies to the right edge too, of course). Figure A depicts these angles, as well as the depth of the ball, Z, the interocular distance, iod, and the retinal visual angle subtended by the ball (same for each eye because ball always lies equidistant from each eye), θ_I :

$$\theta_I = {}_L\theta_E - {}_L\theta_C$$

$$\theta_I = {}_R\theta_E - {}_R\theta_C$$

Using basic trigonometry,

$$\arctan\left(\frac{iod}{2Z}\right) = {}_L\theta_V - {}_L\theta_C$$

$$\arctan\left(\frac{iod}{2Z}\right) == {}_R\theta_C - {}_R\theta_V$$

We define the relative disparity between each eye's image of the ball's edge as:

$$\theta_{\Delta} = {}_L\theta_E - {}_R\theta_E$$

Combining equations we get:

$$\theta_{\Delta} = {}_L\theta_V - {}_R\theta_V - 2\arctan\left(\frac{iod}{2Z}\right)$$

Clearly the relative disparity for the ball's edge provides no direct information about the ball's physical size. From this equation it is obvious that if the observer knows the vergence angles, $\{ {}_L\theta_V , {}_R\theta_V \}$, or is fixating any point along the midline between the eyes (perpendicular to the interocular distance) such that, ${}_L\theta_V = {}_R\theta_V$, then it is possible to use the retinal disparity to solve for Z .

The above result holds for the ball when in the mid-sagittal plane. Our ball did oscillate slightly out of the mid-sagittal plane a maximum of 2.5 deg, which induces a relatively disparity cue of about 0.02 deg as the ball's size changes, which is at or below known human stereo resolution thresholds [A1].

A1. Farell B, Li S, McKee SP (2004) Coarse scales, fine scales, and their interactions in stereo vision. J Vis 4(6): 488-499
